# Dissipative Particle Dynamics Investigation of the Transport of Salicylic Acid through a Simulated In Vitro Skin Permeation Model

**DOI:** 10.3390/ph11040134

**Published:** 2018-12-05

**Authors:** Daniel P. Otto, Johann Combrinck, Anja Otto, Louwrens R. Tiedt, Melgardt M. de Villiers

**Affiliations:** 1 Research Focus Area for Chemical Resource Beneficiation, Laboratory for Analytical Services, North-West University, 11 Hoffman Street, Potchefstroom 2531, South Africa; 2 Centre of Excellence for Pharmaceutical Sciences, North-West University, 11 Hoffman Street, Potchefstroom 2531, South Africa; 21146284@nwu.ac.za (J.C.); anja_otto@hotmail.de (A.O.); 3 Research Focus Area for Chemical Resource Beneficiation, Laboratory for Electron Microscopy, North-West University, 11 Hoffman Street, Potchefstroom 2531, South Africa; louwrens.tiedt@gmail.com; 4 School of Pharmacy, University of Wisconsin–Madison, 777 Highland Avenue, Madison, WI 53705, USA; melgardt.devilliers@wisc.edu

**Keywords:** skin, nitrocellulose membrane, interaction parameter, diffusion, atomistic molecular dynamics, dissipative particle dynamics

## Abstract

Permeation models are often used to determine diffusion properties of a drug through a membrane as it is released from a delivery system. In order to circumvent problematic in vivo studies, diffusion studies can be performed in vitro, using (semi-)synthetic membranes. In this study salicylic acid permeation was studied, employing a nitrocellulose membrane. Both saturated and unsaturated salicylic acid solutions were studied. Additionally, the transport of salicylic acid through the nitrocellulose membrane was simulated by computational modelling. Experimental observations could be explained by the transport mechanism that was revealed by dissipative particle dynamics (DPD) simulations. The DPD model was developed with the aid of atomistic scale molecular dynamics (AA-MD). The choice of a suitable model membrane can therefore, be predicted by AA-MD and DPD simulations. Additionally, the difference in the magnitude of release from saturated and unsaturated salicylic acid and solutions could also be observed with DPD. Moreover, computational studies can reveal hidden variables such as membrane-permeant interaction that cannot be measured experimentally. A recommendation is made for the development of future model permeation membranes is to incorporate computational modelling to aid the choice of model.

## 1. Introduction

Transdermal delivery provides several advantages over other routes of drug administration. These include the avoidance of regular oral dosing, circumvention of first-bypass hepatic metabolism and improvement of patient compliance [1]. Diffusion studies performed in skin models aid quality control and formulation of transdermal delivery systems [2,3]. However, the selected membrane models are often only useful under certain conditions [4] and could affect the evaluation of bioequivalence of generic transdermal products [5,6,7].

Transdermal diffusion studies are often conducted by in vitro studies, utilizing excised skin tissue or (semi-)synthetic membranes. In vitro tests are preferred to in vivo test methods that often demonstrate large intra- and intersubject variation, high cost and ethical issues. The Franz cell in vitro method [8] is well known and overcame some in vivo difficulties. In vitro models are however, also problematic since some models cannot account for drug metabolism in the skin. Additionally, large intra- and intersubject variability of donor skin occurs and tissue preparation can damage the skin membrane [9]. Although skin models are the most realistic model to evaluate transdermal studies, it is difficult to find donors and skin is therefore, a scarce resource. Furthermore, ethical approval is often a difficult hurdle to cross.

In vitro test membranes, synthetic and semisynthetic, are skin-mimicking in vitro model membranes and include silicone-based membranes [7,10,11,12], cellulose-based membranes [2,3,13,14] and chitosan membranes [15,16]. Silicone membranes -limit the diffusion rate while porous cellulose and polysulfone membranes were proven to be non-limiting [17]. A number of polysaccharides were previously modelled by AA-MD. Ion conduction through chitosan membranes was reported for which twelve chitosan monomers were coupled to form a chain model [18]. The chain structure of cellulose II was investigated for which sixty glucose residues were used as a model cellulose chain [19]. Deformation of regenerated cellulose fibres was investigated by AA-MD, however the chain length was not reported [20] and a trinitrocellulose model was used to study the material as a propellant. Trinitrocellulose was used as the closest model reference to our study [21].

Numerous factors [22] should be considered before drawing conclusions based on in vitro models. Diffusion through biological membranes is a universally recognized thermodynamic process [22,23,24] and greatly affects drug permeation through membranes [25,26]. Finite [27] (typical dosage) or infinite [26] (saturated) dosing can determine the thermodynamic driving force for diffusion [28,29]. Thus, the observation of steady-state conditions in the transport process from which flux is determined is affected by saturation. Model membranes should also have a predictable interaction, or lack thereof, with the drug since it could modify compartment partitioning [30]. To mimic skin barrier properties, silicone-type membranes are preferred [31].

Several types of modelling have been applied to explain drug transport. Mathematical modelling has been used to determine diffusion and permeation parameters from experimental data [32,33,34]. Mathematical models aim to improve predictability of drug release, permeation and penetration of drugs, optimize formulation and release and estimate toxicology [32]. Structural changes in skin components that occur during diffusion have been modelled for phospholipid and ceramide layer in the stratum corneum [35] and solvent-solute-membrane interactions [36,37,38]. 

Few studies modelled large scale biological membrane transport via dissipative particle dynamics (DPD) simulations regarding drug delivery. DPD is a computational method of coarse-graining. This means that individual atom properties are transferred to representative particle or coarse-grained bead which have the properties of a number of atoms. DPD allows for a significant lowering of the degrees of freedom in a computation system and facilitates longer simulations of more expansive systems, whilst still accurately reflecting atomistic simulations. The properties that coarse-grained beads assume are typically derived via AA-MD simulations. Effectively, AA-MD information is mapped onto collective, coarse-grained DPD particles [39,40,41]. Nanoparticle transport through cell membranes has been modelled [42], interaction and penetration of charged dendrimers through lipid bilayers were modelled [43] and lipid membranes have been modelled [44]. Pharmaceutical DPD modelling aided designing of polymeric nanomicelles [45], studied the microstructures of pH-responsive amorphous solid dispersions [46] and studied quercetin release from solid dispersions [47]. A number of molecular dynamics studies were also reported that simulated apply to skin simulation. Prediction of the permeation of drugs through the skin was modelled by molecular dynamics [48]. The permeation of water through a lipid bilayer, that was representative of the stratum corneum, was also successfully modelled by AA-MD [49]. The permeation of various molecules through the skin lipid bilayer was also recently reported [50].

Here, it is shown that the finite dose approach may produce flux results that is difficult to explain if the possible effect of the membrane on drug transport is not considered. Salicylic acid is one the most commonly employed drugs in topical delivery systems. It provides a simple model for experimental transport studies and also for computational studies. It is a highly water-soluble drug and its pH-pK_a_-dependent state of ionization makes it an interesting substance to confirm or exclude interactions with model membranes which might in turn also demonstrate different states of ionization. In this study, experimental investigation of the transport of salicylic acid through a commonly used model membrane, nitrocellulose, is presented. It is subsequently shown that a computational approach can be used to simulate the experimental findings. Computational modelling is suggested as means of revealing drug transport through a model membrane. Computational simulation is suggested as a tool to aid selection of membrane models. The experimental and DPD model results confirmed each other.

## 2. Results

### 2.1. Experimental Results

#### 2.1.1. Solubility and Release Studies

The solubility, swelling degree and degree of ionization of salicylic acid at different pH values are summarized in Table 1.

It was observed that an increase in solubility of salicylic acid occurred with an increase in pH that confirmed experimental observations [51]. The increase in solubility with increasing pH could be explained by a rise in the degree of ionization of salicylic acid (as indicated in Table 1) since the pK_a_ of salicylic acid is ~2.97.

The cumulative release of salicylic acid per surface area was plotted against the square root of time, Equation (1), which is the simplified Hiquchi model [52]:

(1) $$f_{t}=K_{H}t^{1/2}$$

where f_t_ is the cumulative amount of salicylic acid released per surface area, K_H_ is the Higuchi dissolution constant, that is, release rate and t^1/2^ represents the square root of time. K_H_ was calculated from the linear part (t_1h_–t_12h_) of the curve. K_H_ and corresponding regression coefficients as well as the cumulative amount of released salicylic acid in 12 h are presented in Table 2.

**Figure 1:**
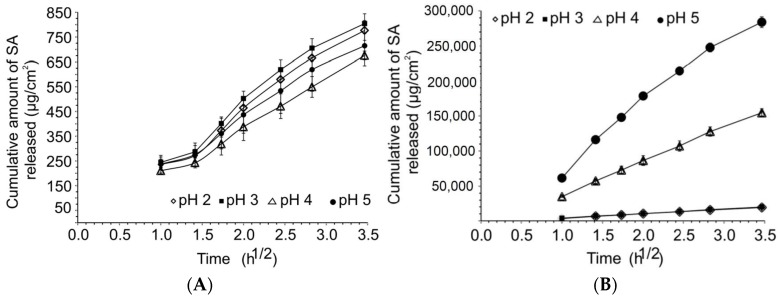
(**A**) Cumulative release of salicylic acid from unsaturated solutions at various pH levels. (**B**) Cumulative release of salicylic acid from saturated solutions at various pH levels. (The graph of pH 3 overlays the graph of pH 2).

As seen in Figure 1 and the regression coefficients in Table 2, the amount of salicylic acid released per surface area showed a linear relationship with the square root of time (R^2^ ≥ 0.99) for all the drug-saturated or unsaturated buffer solutions.

Unsaturated solutions did not show statistically significant differences (*p* > 0.05) in the release rate or cumulative amount of salicylic acid released over 12 h between the different pH values although the degree of swelling was high for saturated and unsaturated solutions at different pH levels, there was no clear relationship with the cumulative release in different buffer solutions. The cumulative release remained consistent for the unsaturated solutions despite differences in the degree of swelling. Furthermore, the pH of the donor solutions at all pH values remained stable over the 12 h release study for both saturated and unsaturated solutions. The insignificant difference in the cumulative release from the unsaturated solutions suggested that charged salicylic acid might interact with the charged nitroso groups of the nitrocellulose membrane. This resulted in some saturation of membrane pores that prevented significant increases in cumulative release into the acceptor phase.

In case of the saturated solutions, the saturation of pores will still occur; however, an equilibrium level of saturation is reached after which cumulative release of the non-interacting charged salicylic acid will increase. This increase is in accordance with the pH of the buffer solution therefore, the degree of ionization. In buffers where the pH is lower than the pK_a_, the drug is relatively less soluble in the medium. This explains the lower cumulative transport of the neutral drug molecules, although it was comparatively higher in the saturated systems than in unsaturated systems. The solubility-pore saturation interplay could be one of the mechanisms to explain the difference in transportation of salicylic acid from saturated and unsaturated solutions.

#### 2.1.2. Membrane Morphology

No differences in membrane structure were observed by SEM imaging for the membranes that were subject to donor solutions of salicylic acid at various pH values. Therefore, membrane integrity was maintained over the range of pH values (Supplementary Materials S3, Figure S1).

### 2.2. Computational Studies

#### 2.2.1. AA-MD Computational Studies—Interaction Parameters

Uragami et al. [53] found that the interaction between nitrocellulose and solvents or solutes played an important role in the permeation of actives through the membrane. A higher cumulative release of the tested analytes was found for cellulose membranes compared to nitrocellulose membranes. This observation pointed to different interactions between the functional groups on the different membranes with the solvent and solute. χ_ij_ and *E*_mix_ values were calculated (Supplementary Materials S4, Figure S2). χ_ij_ indicated significant differences in the interaction between charged and non-ionized species as seen in Figure S4. Two populations of interactions could be classified. Interactions between the charged salicylate species are markedly stronger than for the interactions of neutral salicylic acid. Significant miscibility of salicylate and the other species were indicated by virtue of negative χ_ij_ values. The unionized species illustrated immiscibility seen from positive χ_ij_ values., the χ_ij_ for various bead pairs is given for the temperature range 273–373 K in Supplementary Materials S5, Figure S3, showing no significant change over this temperature range. 

#### 2.2.2. AA-MD—The Choice of the Polymer Chain Length

Choosing an appropriate, representative chain length can ensure optimal computation duration as well present realistic physical properties of the nitrocellulose membrane. The CED and simulation cell density were monitored as criteria to determine the suitability of the oligomer length as representative chain length for a polymer (Supplementary Materials S6, Figure S4).

It was observed that the CED and density values were virtually invariant as observed for different oligomers. The maximum difference in CED between the oligomers was ~5.4%, ranging between 4.05–4.55 × 10^8^ J/m^3^. The density value of all the chain models deviated by a maximum of~2.6% from the target density of 1.49 g/cm^3^. This density was chosen from the closest model reference that was found in literature, for example, trinitrocellulose propellant model studies [21]. A 13*mer* (26 monosaccharides) chain was chosen as best representative oligomer of the polymer chain and its density deviated by only ~1.4% from the target density of 1.49 g/cm^3^, supporting the choice of a 13*mer* chain as model.

#### 2.2.3. DPD Simulations

**Figure 2:**
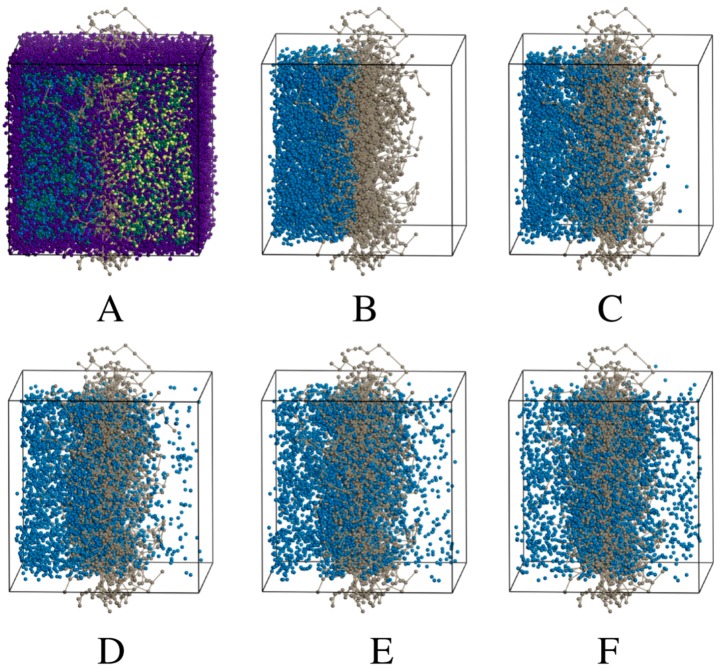
(**A**), the total simulation box containing neutral salicylic acid only with wall beads (purple), nitrocellulose (grey), salicylic acid (blue), water (green) and propylene glycol (yellow). Only nitrocellulose and salicylic acid beads are shown at different times as multiples of τ during the trajectory simulation; (**B**) 1 τ, (**C**) 75,000 τ, (**D**) 250,000 τ, (**E**) 500,000 τ, (**F**) 1,000,000 τ.

**Figure 3:**
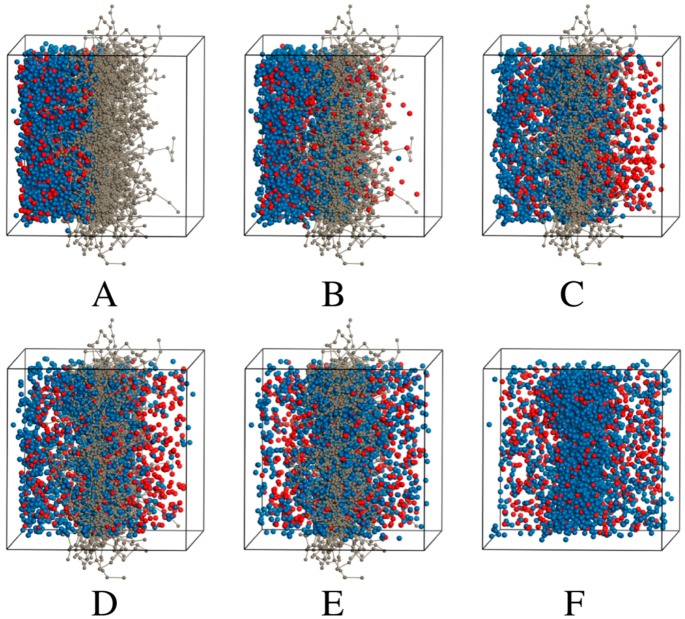
A simulation box of 1:4 charged:neutral drug beads. Nitrocellulose (grey), neutral salicylic acid (blue), charged salicylate (red). Only nitrocellulose, neutral and charged drug beads are shown at different multiples of τ during the trajectory simulation; (**A**) 1 τ, (**B**) 25,000 τ, (**C**) 187,000 τ, (**D**) 250,000 τ, (**E**) 500,000 τ and (**F**) 1,000,000 τ (nitrocellulose is not shown).

**Figure 4:**
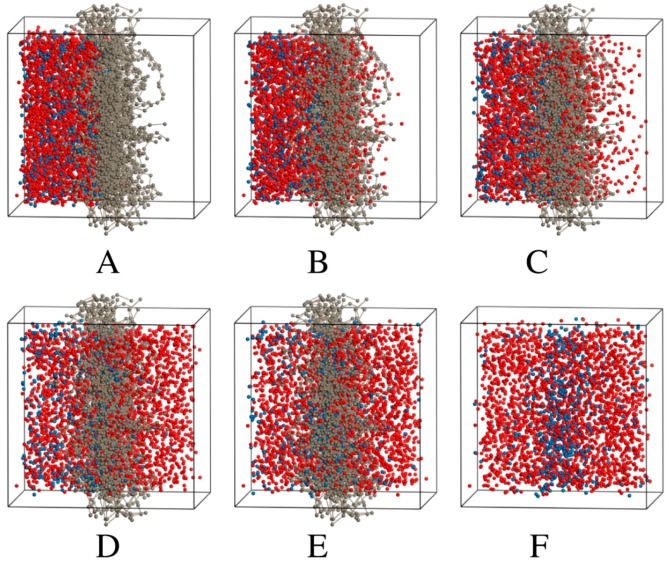
A simulation box of 4:1 charged:neutral drug beads, with wall beads (purple), nitrocellulose (grey), salicylic acid (blue) and salicylate (red). Only nitrocellulose, neutral and charged drug beads are shown at different multiples of τ during the trajectory simulation; (**A**) 1 τ, (**B**) 7500 τ, (**C**) 25,000 τ, (**D**) 125,000 τ, (**E**) 250,000 τ and (**F**) 500,000 τ (nitrocellulose is not shown) (see electronic version for colour figure).

The trajectories of the various simulations (Figure 2, Figure 3 and Figure 4) show snapshots at various time steps in the simulation of selected models. Additional models are demonstrated in Supplementary Materials S7, Figures S5–S7). All simulation boxes contained ~29,000 beads.

The DPD models all showed that the charged drug beads diffused faster than neutral beads. Some charged particles remained in the membrane in all the models. However, as seen from the models, a portion of neutral particles assembled close to or inside the membrane.

Eventually, the neutral particles are transported to the acceptor side and this can be ascribed to the concentration gradient. Since the ionized (charged) particles are more water soluble than the unionized particles, they are transported faster and to a larger extent.

**Figure 5:**
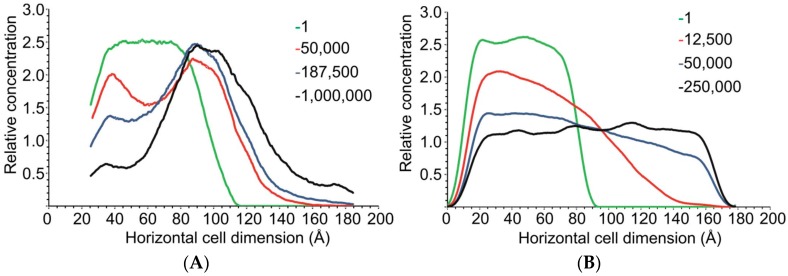
(**A**) Concentration profile snapshots at various time step values (τ, shown in the legend) for a fully neutral diffusion system. (**B**) Concentration profile snapshots at various time step values (τ, shown in the legend) for a fully charged diffusion system.

The number of time steps, τ taken to reach equilibrium transport clearly became less as the amount of charged drug beads in the system increases relative to neutral beads. For the fully neutral system shown previously in Figure 2, the beads reach equilibrium in both compartments only after 1,000,000 τ. The fully charged system shows that charged molecules are already approaching equilibrium after 50,000 τ. Concentration profiles of the fully charged and neutral systems are plotted in Figure 5.

Figure 5A indicated that some salicylic acid accumulates in the nitrocellulose membrane or close to the membrane in the region of 80–100 Å. This again reflects that the χ_ij_ between nitrocellulose and charged salicylate particles would favour transport, with a fraction of the charged drug interacting with the membrane before the excess will diffuse to the receptor compartment. The neutral beads will eventually diffuse once the diffusion gradient forces particles to the acceptor side. 

Figure 5B illustrates that the charged drug particles reach equilibrium between the compartments as seen from the flat top curve achieved at 250,000 τ.

**Figure 6:**
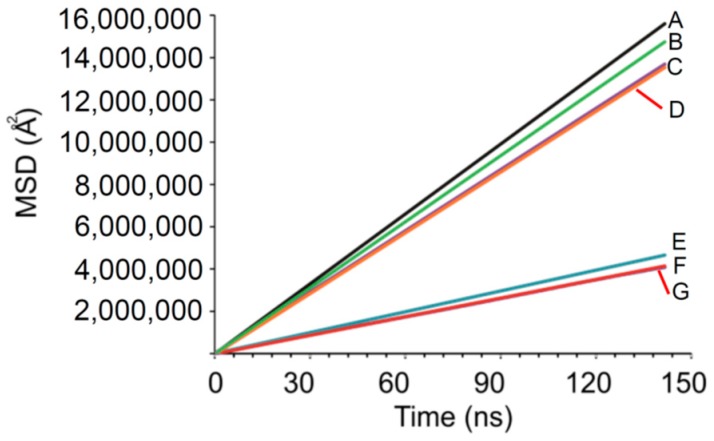
MSD plots of charged (top group curves) and neutral salicylic acid species (bottom group curves) for various compositions as mentioned in the text. A is the system where only charged salicylate beads are present, B charged to neutral salicylic acid beads in 4:1 ratio, C is the system with charged to neutral salicylic beads in 1:1 ratio, D is the 1:4 charged to neutral bead system, E is 1:99 charged to neutral salicylic/particles, F is the fully neutral salicylic acid model.

The root mean square displacement (MSD) of charged and neutral beads was also determined for fully neutral and charged systems as well as for their ratios of 1:4, 1:1 and 4:1. MSD gives an estimation of the rate of diffusion of the species as function of time. Figure 6 illustrates the findings.

Figure 6 depicted two groupings of continuances of linear curves with R^2^ > 0.9999, indicating the free diffusion regimen was reached. The top group of curves indicates the MSD for charged salicylate beads and the bottom group that of the neutral salicylic acid particles. Differences in the slopes for each curve within a group were seen. The charged group showed a slope of ~101.7 ± 7% (% CV) and the neutral group a slope of ~30.4 ± 6%, indicating that charged particles diffused 3.33 times faster than neutral ones. These rates are not physically accurate since it is known that DPD simulations either can under- or overestimate the values of measured diffusion constants [54]; however, the relative diffusion trends are still seen. These curves also illustrate that the beads in the model demonstrated a consistent behaviour irrespective of the proportion of charged and neutral particles. Charged salicylate always diffused faster than neutral salicylic acid. This validates the DPD model as a discriminatory mechanism to study the effect of pH and therefore the charge of particles, on rates of diffusion. The diffusion rates again correspond to the calculated χ_ij_ values.

A question that arose was if the diffusion of propylene glycol in the model from the acceptor compartment to the acceptor compartment would be different for each of the systems with different ratios of charged and neutral beads. A concentration profile was constructed for propylene glycol; at the start and end of the simulations, that is, at 0 τ and 1,000,000 τ for selected systems and depicted in Supplementary Materials S8, Figure S8.

Propylene glycol diffused to the same extent for the 1:1 and fully charged systems. Approximately one third of all the propylene glycol diffused from the donor to acceptor side. It can be deduced from the more favourable χ_ij_ value of propylene glycol and charged species that more propylene glycol would be accommodated by charged particles if propylene glycol were also present on the acceptor side. Conversely, due to the diffusion gradient, propylene glycol diffused to the acceptor chamber in the fully neutral system. The diffusion of propylene glycol is much slower as was expected from the less favourable χ_ij_ value of propylene glycol and neutral particles. 

## 3. Discussion

### 3.1. Release, Partition and Morphology Studies

No statistically significant differences were observed in release of salicylic acid from the unsaturated buffer solutions at different pH values. Conversely, the release of salicylic acid from saturated solutions increased proportionally with an increase in pH.

No correlation was found between the release of salicylic acid and the degree of swelling. No differences in the structure of nitrocellulose membranes were observed at the different pH values, indicating that the pore size of the membrane was independent of pH.

Our experimental release study of unsaturated salicylic acid solutions contradicted the results obtained by Asman et al. [55] where the release of salicylic acid from unsaturated aqueous donor phases through poly(vinyl alcohol-*g*-itaconic acid) and poly(vinyl alcohol) membranes increased with decreasing pH. Our study found no statistically significant release differences for unsaturated solutions of salicylic acid through nitrocellulose membranes.

However, different explanations were given by Asman et al. [56], investigated the effect of the model membranes that they used. The decrease in release through poly(vinyl alcohol) membranes, with increasing pH, was related to a decline in the swelling of the membrane, limiting the free volume in the membrane that is available for transport. The release results, obtained from poly(vinyl alcohol-*g*-itaconic acid) membranes were attributed to electrostatic repulsion between the anionic carboxylate groups of both itaconic acid and salicylic acid. This led to the conclusion that only the unionized molecules of salicylic acid determined the release of salicylic acid through the poly(vinyl alcohol-*g*-itaconic acid) membranes.

However, Asman et al. [56], also found contradictory results when poly(vinyl alcohol-*g*-acrylamide) membranes were used. In that study, release increased proportionally to an increase in pH. This was ascribed to an increase in release of salicylic acid that contributed to the bonding of ionized salicylic acid to the membrane on the receptor side by hydrogen bonding.

Furthermore, it was seen that the percentage salicylic acid released at pH 2.10 and 7.40 were similar. This similarity was explained by the hydrolysis of the amide groups of the poly(vinyl alcohol-*g*-acrylamide) membranes at pH 2.10 that resulted in higher degrees of swelling.

Both studies by Asman et al. [55,56], showed contradictory results, depending on the membranes used and it could be concluded that the membranes as well as the method affected the release. It does not invalidate these experimental models though. They do however; show that the results of a flux study on the same drug depend strongly on the experimental conditions and the chosen model membrane. This is also the case for the experimental results in this study.

### 3.2. Computational Studies

#### 3.2.1. AA-MD Computational Studies—Chain Length and χ_ij_

The 13*mer* nitrocellulose model of this study has shown an accurate agreement concerning material density for the chosen model oligomer close to the value of 1.49 g/cm^3^ [21] as well as the minimal variation in CED. In this study, the final production AA-MD trajectories were significantly longer than the picosecond runs performed previously for polysaccharide models (see introduction).

As far as χ_ij_ is concerned, it was not surprising that the charged, dissimilar pairs would show stronger, attractive interactions than compared to the situation where only one component of the pair was charged. Furthermore, the neutral species showed weaker interaction in their dissimilar pairs than for the electrostatically interacting beads.

It is advocated that AA-MD simulations could be used in future studies to predict how an experimental system would perform. Using χ_ij_ screening, the choice of experimental membrane could be made and possibly predict the interactions between the membrane and transportable species. In this way, potential transport-limiting membrane effects could be identified. The CED values that are derived from the AA-MD simulations are converted to solubility parameters. The solubility parameters are then factorized in dispersive and electrostatic components that could further aid the choice or design of the model membrane in relation to the drug solubility parameters.

#### 3.2.2. DPD Computational Studies

DPD simulation is a powerful tool to visualize the diffusion of various components through the membrane. Since the AA-MD methods are limited to lower timescale simulations and are computationally expensive, DPD provides the bridge to access longer simulations.

DPD simulations of this study illustrated that the atomic scale properties were transferred accurately to the DPD scale since the bead-based models exhibited the expected diffusion behaviour as predicted from AA-MD. DPD simulations showed that bead interactions could be modelled. Furthermore, modelling also demonstrated the difference between the transport rates in terms of τ for charged and neutral particles. 

The models also indicated the presence of charged drug particles that associate with the membrane, before the remaining bulk will be transported from the donor to the acceptor chamber once saturation of the membrane was reached.

DPD could also discriminate between neutral and charged particles in terms of MSD versus time and showed that neutral and charged species diffusion behaviour was the same in different proportions of these particles. The charged particles diffused faster than the neutral particles in all models.

Therefore, the DPD model did not introduce artefacts due the presence of either neutral or charged particles or their combinations.

## 4. Materials and Methods

### 4.1. Materials

Miglyol 812 N^®^ was donated by Cremer (Hamburg, Germany). Salicylic acid (>99%) was obtained from (St. Louis, MO, USA). Potassium chloride and citric acid anhydrous were purchased from Sigma Aldrich (Johannesburg, RSA). Sodium dihydrogen orthophosphate, disodium hydrogen orthophosphate anhydrous, propylene glycol, 1 N hydrochloric acid, 1 N sodium hydroxide solution and methanol were purchased from ACE Chemicals (Johannesburg, RSA). Acetonitrile (LiChrosolv^®^) and glacial acetic acid were purchased from Merck Chemicals (Johannesburg, RSA).

### 4.2. Experimental Methods

#### 4.2.1. Aqueous Phase Preparation

Citric acid-phosphate buffer solutions were prepared at pH 2.00, pH 3.00, pH 4.00 and pH 5.00. Unsaturated salicylic acid solutions were prepared in citrate-phosphate buffers at various pH and stirred for 12 h to yield a concentration of 1 mg/mL. Saturated salicylic acid was also prepared using the buffer solutions. The pH value of these solutions was adjusted to the required value if needed.

#### 4.2.2. Solubility Determination of Salicylic Acid

Saturated salicylic acid solutions were prepared in the buffer solutions. Salicylic acid was added to 20 mL buffer solution until it did not dissolve any more. The excess settled at the bottom. The medium in which solubility was determined was the same as the receptor phase that was employed for the permeation studies. The solutions comprised of phosphate buffer and propylene glycol (1:1, *v*/*v*) at pH 7.40. Solutions were stirred at 37 ± 1 °C with continuous adjustment of the pH to the required value [57]. After 24 h, the solutions were filtered through syringe filters with a pore size of 0.45 µm (Agela Technologies Inc., Newmark, New York, NY, USA, USA) and immediately diluted 1:100 with methanol and analysed by HPLC. UV detection was employed at 236 nm (Section 4.2.8).

#### 4.2.3. Determination of Percentage Ionized Salicylic Acid

The percentage of ionized salicylic acid in the solutions was determined using the Henderson-Hasselbalch, Equation (2) [58,59,60]:

(2) $$\mathrm{pH}-{\mathrm{pK}}_{a}=\log\frac{\left[A^{-}\right]}{\left[\mathrm{HA}\right]}$$

where pK_a_ is the logarithmic acid dissociation constant, (for salicylic, pK_a_ ~2.97), [A^−^] is the molar concentration of the conjugate base and [HA] is the molar concentration of the undissociated weak acid.

#### 4.2.4. In Vitro Release Study

Salicylic acid release was studied using Franz type diffusion cells with a diffusion area of 1.13 cm^2^ exposed by nitrocellulose membranes (0.2 µm pore size, Whatman, Dassel, Germany). Four cells were used per solution. Temperature was maintained thermostatically at 37 ± 1 °C for 12 h. 

Nitrocellulose membranes were soaked overnight in buffer/propylene glycol (PG) (1:1, *v*/*v*) and also in pure buffer solutions. The receptor compartment was filled with 2 mL preheated, degassed receptor fluid and allowed to equilibrate before adding the donor phase by stirring at 750 rpm. The donor compartment was subsequently filled with 1 mL of salicylic acid solutions prepared in the buffers. Samples were taken at 1, 2, 3, 4, 6, 8 and 12 h and comprised of the full 2 mL of the receptor phase. After sampling 2 mL fresh receptor phase was immediately replenished in the receptor compartment. Samples were prepared for HPLC and analysed by UV detection at 236 nm (Section 4.2.8). pH was monitored at the time of sampling.

After completion of the release study, the nitrocellulose membranes were removed, dried and imaged by SEM. Two Franz cells were also exposed to blank donor buffer solutions. After 12 h, the blank membranes were removed, dried and imaged by SEM.

#### 4.2.5. Statistical Analysis

Data were analysed by one-way ANOVA using STATISTICA^®^ (StatSoft Inc., Tulsa, OK, USA). Tukey’s HSD (Honestly Significant Difference) test was performed to compare the release at different pH values with each other at a significance threshold of *p* < 0.05.

#### 4.2.6. Degree of Swelling

Membranes were removed from the cells and excess droplets were removed and the membrane was immediately weighed (wet mass). In addition, an untreated, dry nitrocellulose membrane was weighed (dry mass). The degree of swelling was calculated according to Equation (3):

(3) $$\%\;\mathrm{swelling}=\frac{w-w_{0}}{w_{0}}$$

where W is the wet mass after swelling and W_0_ the dry mass of the membrane before swelling.

#### 4.2.7. Scanning Electron Microscope (SEM)

Membranes sections were mounted on double-sided carbon tape. Au/Pd (67/33) was sputtered on the membranes forming a 15 nm coating. Membranes were imaged using a FEI Quanta FEG 250 scanning electron microscope at 5 kV under high vacuum mode.

#### 4.2.8. HPLC-UV method

An Agilent^®^ 1100 Series HPLC system was used (Agilent Technologies, Palo Alto, CA, USA) using a reversed phase C18-2 column (150 × 4.60 mm, 5 µm particle size) (Venusil XBP Agela Technologies, Wilmington, DE, USA). Temperature was controlled at 25 ± 1 °C.

Degassed mobile phase (*v*/*v*) consisted of 1% acetic acid, 45% acetonitrile and 54% Milli-Q^®^ water. Flow rate was set at 1 mL/min and analysed at 236 nm. Samples were injected in duplicate.

### 4.3. Computational Modelling Methods

All computational modelling was performed using the Materials Studio 6.1 (Accelrys Software Inc., San Diego, CA, USA) suite of packages. The modules used for modelling all refer to the proprietary names of the modules.

#### 4.3.1. All-Atomistic Molecular Dynamics (AA-MD) Theory

The theoretical background of AA-MD is discussed in the Supplementary Materials S1. The references are listed in the supplementary text as well as in the references below.

**Figure 7:**
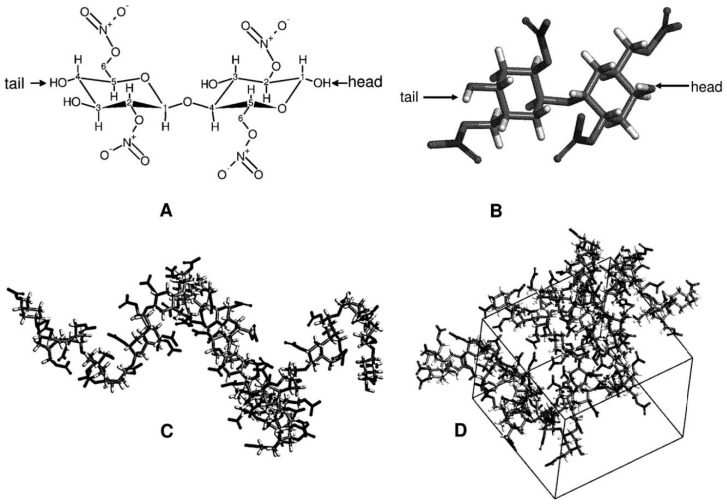
(**A**) the 2,6-dinitroester-pyranose disaccharide of nitrocellulose that was used to simulate the nitroester with a degree of substitution of 2 (the carbon atoms are denoted by numbers). Head-to-tail addition was performed for polymer formation. The *β*-D(1→4) glycosidic bond is also shown in the disaccharide structure. (**B**) the geometrically optimized structure of the disaccharide repeat unit, (**C**) an example of a geometrically optimized 30*mer* chain and (**D**) a periodic simulation box packed with 10*mer* chains.

Figure 7 illustrates the model that was developed and used for AA-MD. AA-MD is a thermodynamics modelling process which has been used to successfully study the thermodynamics of diffusion and therefore applicable to transdermal diffusion studies.

#### 4.3.2. AA-MD Methodology

At the time of writing, no parameters for an AA-MD study on nitrocellulose were reported. It is therefore, imperative to determine the appropriate length of a model polymer chain to represent the bulk polymer chain. Unsubstituted cellulose has a syndiotactic tacticity [61]. It was therefore, assigned to nitrocellulose. Nitroester functional groups were substituted for the 6-hydroxyl and 2-hydroxyl substituents of cellulose to produce nitrocellulose molecules [62] with a degree of nitroesterification of 2, typical for nitrocellulose filter materials [63].

Subsequently, nitrocellulose chains with 1–40 disaccharides, designated as 1*mer*–40*mer*, were constructed. These models were optimized geometrically with COMPASS force field [64]. Dispersive force summations were applied with a cut-off distance of 12.5 Å. 

The energy tolerance was 4 × 10^−4^ kJ/mol, force tolerance was 0.02 kJ/mol/Å and displacement tolerance was 5 × 10^−5^ Å. The number of iterations was limited to 1 × 10^5^ steps.

Optimized oligomers were subsequently packed in cubic periodic simulation boxes. Thirty disaccharide chains could be packed successfully. Five 1–20*mer* chains could be packed successfully. Four 30- or 40*mer* chains could be packed successfully. All periodic cells were packed to a target density for nitrocellulose of 1.49 g/cm^3^. Box dimensions were set large enough to avoid self-interaction of the chains [65]. Ten optimized simulation boxes of each oligomer cell packing were generated and the lowest energy box was selected for further simulations. All the AA-MD simulations were conducted with time increments of 1 fs.

The boxes underwent simulated annealing [50,66,67,68] via an NVT (constant number of particles, volume and temperature) canonical, ergodic ensemble, employing the Andersen thermostat [69], to ensure initial energy relaxation. Five annealing cycles were simulated at temperatures ranging between 300–500 K for heating and vice versa for cooling in 40 K steps (5 heating and 5 cooling steps). 10,000 dynamic steps were followed for each heating and cooling ramp.

After annealing, the boxes were relaxed further by alternating NVT and NPT (constant number of particles, constant pressure and constant temperature) AA-MD runs of 100 ps employed the Nosé thermostat [70] at 310 K (experimental temperature). The NPT runs employed the Berendsen barostat [71] at ~0.1 GPa. After each NPT and NVT cycle, cell dimensions and cell density were analysed. Once these parameters were consistent, a production run of 5 ns NPT run was performed.

Cohesive energy density (CED) was calculated from the production run for each model. Once the CED assumed a constant value, this was identified as the minimum oligosaccharide chain length that will represent the CED of long chain polysaccharides [72,73].

#### 4.3.3. Dissipative Particle Dynamics (DPD) Simulation Theory

The theory of DPD simulations is discussed in the Supplementary Materials S2. The references are listed in the supplementary text as well as in the references below.

#### 4.3.4. DPD Methodology

The average values for χ_ij_ were calculated from the AA-MD production runs and converted to reduced DPD interaction parameters [73], *a*_ij_, that was assigned to the beads as seen in Table 3.

χ_ij_ was <<0.5 and an average bead volume of 170 Å^3^ and a cut-off radius, *r*_c_ of ~9 Å, were calculated by the Connolly method [76,77]. A simulation box of 20*r*_c_ × 20*r*_c_ × 20*r*_c_ was used as template and surrounded by the wall beads. Simulation boxes with different ratios of the charged to neutral beads were evaluated at ratios of 1:1, 1:4 and 4:1. Trajectories of the different components were evaluated during a preliminary run and it was observed that a million steps could be used at a simulation time step of τ = 0.001, corresponding to ~5 fs. The short time step was chosen to ensure high resolution of the integration grid [50] with respect to t. The time scale falls within measured time scale parameters for a diffusion process [78,79]. DPD trajectories were constructed as a function of time by integration of the Newtonian laws of motion by Equation (4) [80,81]:

(4) $$\frac{{\mathrm{dr}}_{i}}{\mathrm{dt}}=v_{i},\frac{{\mathrm{dv}}_{i}}{\mathrm{dt}}=f_{i}$$

where, r_i_ is the position of the bead i, t is the time point at which the trajectory of bead i is calculated, v_i_ is the velocity of i at t and f_i_ is the derived force experienced by i at t. f_i_ can be described further according to Equation (5) [75,81]:

(5) $$f_{i}=\sum_{i\neq j}\left(F_{\mathrm{ij}}^{C}+F_{\mathrm{ij}}^{D}+F_{\mathrm{ij}}^{R}\right)$$

where i and j are dissimilar beads, F^C^, F^D^ and F^R^ represent the conservative, soft-repulsion force between the beads, the dissipative force due to viscous drag or friction between beads which depends on their position and the random force, respectively. The random force is determined by the amount of energy input at t, by the barostat and thermostat to ensure that the energy in the system does not show a too large fluctuation over time. Summation of forces in the simulation box is performed over *r*_c_.

In case of polymer membranes, or other CG models where many beads are connected, a spring constant determines the stiffness of the connection between the beads by Equation (6) [81]:

(6) $$F_{i}^{S}=\sum_{i}{\mathrm{Cr}}_{\mathrm{ij}}$$

where F^S^ is the total spring force summed over the connected beads i and j, C is the spring constant with a value of 4 which adequately describes polymers and other organic molecules [75]. Large spring constants indicate an increase in chain rigidity.

Geometry optimizations were performed on each simulation box until the convergence limits of 0.004 kJ/mol and a force of 0.5 N were met. Density of the simulation boxes was monitored up to the point of consistency. After optimization, the membrane and wall beads were constrained positionally. This reflected the experimental findings where it was found that the nitrocellulose membrane did not swell nor showed differences in the pores as imaged by SEM (Supplementary Materials S3). Therefore, the water, propylene glycol and drug beads could diffuse whilst the membrane and wall beads remained in position.

## 5. Conclusions

The study revealed that an increase in pH did not significantly affect the amount of salicylic acid that was released from unsaturated solutions. However, the opposite effect was observed for saturated solutions. No effect of the pH of the donor solution on nitrocellulose could be observed, nor any correlation to swelling of the membranes.

The choice of a specific membrane as a model for finite dose diffusion through skin should be made cautiously. This is especially true for quality control studies where a porous membrane such as nitrocellulose could be selected. As shown by the experimental results, the expected effect of pH on drug flux could not be observed in the unsaturated solutions. This study therefore, suggests that a membrane-solute interaction took place that affected the observed diffusion of salicylic acid from unsaturated solutions. This membrane interaction could be absent in the skin and therefore, the model membrane will not reflect the experimental results correctly. We therefore, suggest that modelling could aid the identification of drug-membrane interactions which might not occur, or could occur, in the skin. In general, it is apparent that the nitrocellulose membrane does not accurately reflect the nature of the stratum corneum which is highly lipophilic. Due to the permanent charge of the nitrocellulose nitroso groups, one should not discount the interaction that is possible due to polar groups in the membrane. Numerous hydroxyl groups are present and could interact with polar molecules.

Considering that nitrocellulose is a charged molecule, it is suggested that it should rather be used as a model that mimics interactions between solutes and skin components, especially when finite dose studies are performed. Where saturated solutions of salicylic acid were used, the expected effect of pH on salicylic acid ionization and transport could be observed. It could be attributed to the membrane-solute interaction that resulted in the salicylic acid saturation of the membrane and subsequently the diffusion equilibrium was achieved after salicylic saturation of the membrane was reached.

Computational modelling was also developed to provide a means to study the commonly used in vitro model membrane, nitrocellulose. Computational modelling negates guesswork from choosing a suitable membrane since it is based on fundamental and well-known physical theory. Numerous studies have reported the computed effects of chemicals on skin components such as ceramide; however, very few reports could be found that simulated the in vitro model membranes in the context of skin diffusion studies.

Due to the numerous advantages of in vitro models based on artificial membranes compared to in vivo test subjects, it is suggested that AA-MD and DPD could become additional modelling tools too rationally select model membranes. The modelling can be used either as a predictive tool to select the suitable membrane prior to experimental studies or can provide post hoc insight into the mechanism that resulted in experimental observations. Here we report passive diffusion of both charged and uncharged drug particles, where charged particles diffused much faster than the neutral species. The experiments and computational models confirmed one another.

## Figures and Tables

**Table 1:** Saturated solubility of salicylic acid in the aqueous phases at different pH values. In all cases *n* = 3 and the standard deviation is indicated by ±.

pH	Solubility (mg/mL)	Ionized Salicylic Acid (%)
2.00	15.3 ± 0.9	9.9
3.00	23.0 ± 1.3	51.7
4.00	30.6 ± 1.7	91.5
5.00	>34.9 ± 3.2	99.1

**Table 2:** Average values ± standard deviation (*n* = 4) of *k*_H_, cumulative release in 12 h, % swelling and pH values of the donor phase in nitrocellulose membrane test models.

pH	*k*_H_ (µg/cm^2^/h^1/2^)	R^2^	% Swelling	Cumulative Release at 12 h (µg/cm^2^)	Donor pH *t*_0_	Donor pH *t*_12h_
Unsaturated solutions						
2.00	237.0 ± 18	0.99	68.0 ± 3.5	180.7 ± 7.5	2.1	2.3
3.00	246.4 ± 33	0.99	71.0 ± 8.6	171.3 ± 7.4	3.0	2.9
4.00	197.5 ± 8.3	0.99	60.6 ± 1.6	234.3 ± 38	4.0	3.9
5.00	207.9 ± 22	0.99	63.1 ± 7.1	195.7 ± 8.4	5.0	4.9
Saturated solutions						
2.00	6343.3 ± 658	1.0	>140.4 ± 13	189.0 ± 4.0	2.1	2.6
3.00	6410.3 ± 359	1.0	>96.4 ± 5.0	201.2 ± 12	3.0	3.1
4.00	48,587.3 ± 2240	1.0	>569.9 ± 22	199.1 ± 8.5	4.1	3.6
5.00	89,877.5 ± 3233	0.99	>919.3 ± 25	221.1 ± 3.5	5.1	4.2

**Table 3:** *a*_ij_ values used for DPD simulations.

*a*_ij_	Neutral	Charged	Propylene Glycol	Nitrocellulose	Water	Wall
Neutral	25.00 ^a^					
Charged	2.000 ^b^	25.00				
Propylene glycol	32.64	2.000	25.00			
Nitrocellulose	20.35	2.000	41.04	25.00		
Water	37.49	2.000	26.16	56.90	25.00	
Wall	999 ^b^	999	999	999	999	25.00

^a^ A value for *a*_ij_ > 25 indicates repulsion between beads. Wall beads repelled all the beads within *r*_c_. This ensured that beads remained in the simulation box to improve statistical sampling of their Cartesian coordinates of the beads; ^b^ The Flory-Huggins theory [74] proved that polymer-solvent interaction parameters, χ_ij_~ 0.5 will be miscible. However, the theory does not account for ionic interactions as employed in this study [75]. The χ_ij_ values obtained here were negative for ionic interactions. However, to apply in the simulation *a*_ij_ values below 25 indicate strong miscibility and since χ_ij_ was negative, the values were set to 2 to indicate a strong attractive interaction. Wall beads were set such that they repelled all other particles.

## Supplementary Materials

Available online at http://www.mdpi.com/1424-8247/11/4/134/s1. S1: Background on mixing energy (E_mix_) and interaction parameter (χ_ij_) of binary pairs employing a Monte Carlo method, S2: Background on DPD, Figure S1: SEM images, Figure S2: The *a*_ij_ values calculated for the dissimilar pairs, Figure S3: (A) χ_ij_ for miscible pairs at various temperatures. (B) χ_ij_ for immiscible pairs at various temperatures. H2O refers to water, Figure S4: The CED and density values that were obtained for the various chain lengths of nitrocellulose oligomers. The scatter of values is smaller than would be indicated by the depiction if the magnitudes of the axes are inspected, Figure S5: A simulation box of 1:99 (charged:neutral). Nitrocellulose beads (grey), neutral salicylic acid beads (blue) charged salicylate beads (red). Only nitrocellulose, neutral and charged beads are shown at different times as multiples τ of during the trajectory simulation; (A) 1 τ, (B) 12,500 τ, (C) 25,000 τ, (D) 125,000 τ, (E) 500,000 τ and (F) 1,000,000 τ, Figure S6: A simulation box (1:1 charged:neutral) with nitrocellulose beads (grey), neutral beads (blue) and charged salicylate beads (red) shown at different times in multiples of τ of during the trajectory simulation; (A) 1 τ, (B) 7,500 τ, (C) 25,000 τ, (D) 125,000 τ, (E) 250,000 τ, (F) 1,000,000 τ, Figure S7. (A) the simulation box of 100\% charged beads with nitrocellulose beads (grey), wall beads (purple), propylene glycol beads (yellow) and water beads (green) Only charged salicylate and nitrocellulose beads are shown at different times as multiples τ of during the trajectory simulation; (B) 1 τ, (C) 5,000 τ, (D) 12,500 τ, (E) 25,000 τ and (F) 1,000,000 τ.Figure S8: The concentration of propylene glycol as snapshots taken at τ = 1 (solid lines) and τ = 1,000,000 (dashed lines). C is the abbreviation for charged and N for neutral. The 1:1 system is denoted by 50C50N.
