# Supplementary material for: Dissipative Particle Dynamics Investigation of the Transport of Salicylic Acid through a Simulated In Vitro Skin Permeation Model
Source: Pharmaceuticals (Basel). 2018 Dec 5;11(4):134. doi: 10.3390/ph11040134 (PMC6316323; doi:10.3390/ph11040134)
Supplement: Supplementary file 1 [file pharmaceuticals-11-00134-s001.pdf]

pharmaceuticals

MDPI

Article – Supplementary material

Dissipative particle dynamics investigation of the transport of salicylic acid through a simulated in vitro skin permeation model

Daniel P. Otto [1,*], Johann Combrinck [2], Anja Otto [2], Louwrens R. Tiedt [3] and Melgardt M. de Villiers [4]

[1] Research Focus Area for Chemical Resource Beneficiation, Laboratory for Analytical Services, North-West University, 11 Hoffman Street, Potchefstroom, South Africa; 11333561@nwu.ac.za
[2] Centre of Excellence for Pharmaceutical Sciences, North-West University, 11 Hoffman Street, Potchefstroom, South Africa; johann.combrinck@nwu.ac.za
[3] Research Focus Area for Chemical Resource Beneficiation, Laboratory for Electron Microscopy, North-West University, 11 Hoffman Street, Potchefstroom, South Africa
[4] School of Pharmacy, University of Wisconsin–Madison, 777 Highland Avenue, Madison 53705, WI, USA
* Correspondence: 11333561@nwu.ac.za; Tel.: +27-(0)18-299-2361

Received: date; Accepted: date; Published: date

S1 Background on mixing energy (E_{mix}) and interaction parameter (χ_{ij}) of binary pairs employing a Monte Carlo method

To establish a computational model of the diffusion system, it must first be established how different species in the system will interact with each other. E_{mix} and χ_{ij} are two of the most useful descriptors of species interaction. The determination of these descriptors by a computational approach are described next.

The probability of miscibility of different components has traditionally been described for polymers and apolar solvents by the Flory-Huggins theory [59]. Serious drawbacks of this model are the lack of description of forces other than dispersion forces and that the model only applies to pure components and not to the mutual interactions between different components [60].

Due to the shortcomings of the model, an extended form of the Flory-Huggins model was developed that consider the E_{mix} of binary pairs of different components. Mixing and phase separation can be described by Equation S1 [59]:

$$\frac{\Delta G}{RT} = \frac{\varphi_i}{n_i}\ln\varphi_i + \frac{\varphi_j}{n_j}\ln\varphi_j + \chi_{ij}\varphi_i\varphi_j \quad \text{(S1)}$$

where ΔG is the free energy of mixing, ϕ_i and ϕ_j are the volume fractions of components *i* and *j*, *n* is the degree of polymerization of the components, *R* is the universal gas constant, *T* is the absolute temperature and χ_{ij} is the interaction parameter of the components *i* and *j*. The free energy of mixing, ΔG can be rewritten as E_{mix} and $\chi_{ij}=E_{mix}/RT$.

E_{mix} is calculated from determining the free energy due to the binary interaction of the components *i-i* and *j-j* as pure components or as their mixture *i-j* and *j-i*. Numerous spatial arrangements of the components *i* and *j* are simulated by a Monte-Carlo algorithm and the free energy calculated between these pairs, within a certain cutoff distance, is determined. Each of the spatial arrangements of the pairs will result in a different free energy value for that specific configuration or coordination number of the pair. Finally, E_{mix} can then be expressed as the differences in the average free energies for all possible binary pair configurations by Equation S2:

$$E_{mix} = \frac{1}{2}Z\left(E_{ij} + E_{ji} - E_{ii} - E_{jj}\right) \quad \text{(S2)}$$

Where Z is the number of sampled configurations of the component pairs, E_{ij}, E_{ji}, E_{ii} and E_{jj} are the average free energy of binding for the pair of Z configurations. Here we used $Z = 1 \times 10^6$. Therefore, 1,000,000 atomistic-level Monte Carlo configurations were simulated. The simulation was performed at 310 K, the physical experimental temperature of diffusion studies. Van der Waals surfaces of the molecular fragments that represented the configurations were screened against one another to determine the average E_{mix} with Equation S2 [57,72]. χ_{ij} is then finally derived from E_{mix}.

E_{mix} was determined via AA-MD, employing the COMPASS force field [48]. COMPASS assigns force constants that describe the interaction between atoms in a model system. COMPASS has been well-validated for polymers and organic compounds [48]. COMPASS has been applied successfully to polysaccharide modeling studies. Examples include ion conduction in chitosan membranes [15], the investigation of the chain structure of cellulose II [19], the investigation of molecular deformation of regenerated cellulose fibers under strain [20] and the investigation of trinitrocellulose as propellant [21].

To determine E_{mix} and χ_{ij}, complete molecules of water, salicylic acid (neutral), salicylate (charged) and propylene glycol were used. In case of nitrocellulose, the repeat unit (Fig. 8 in the main text) was utilized as the representative bead fragment.

S2 Background on DPD

The coarse-grained molecular dynamics (CG-MD) of cellulose has been described to account for both crystalline and amorphous allomorphs [84]. CG modeling provides a means to transfer atomic-level physicochemical information to bead level; however, achieving a substantially lower computational cost to perform long time scale calculations [85]. The Martini force field approach has been developed for bimolecular modeling and utilizes a 4-atoms-to-1-bead approach. Therefore, for a polysaccharide, four non-hydrogen atoms are grouped together (ring and glycoside bond atoms) into one bead that contains the average physical information of the group of atoms such as dihedral and torsional angles as well as bond lengths etc. [86].

Although some resolution of a measured response is lost by averaging during CG simulations, excellent approximations of AA-MD is produced for CG models. Recently, three CG models have been developed to describe polysaccharides namely, the M3B model which uses a 3-beads-per monosaccharide approach to preserve ring puckering and torsional angles between monosaccharides [87]. A single-bead-per-monosaccharide model was also reported that accurately described crystalline and amorphous cellulose structures [85]. Yet another development expanded of the Martini force field to describe carbohydrates [86].

DPD is a CG-based technique to perform CG simulations [39,40]. No data could be found for nitrocellulose concerning DPD parameters and therefore, Blends (Materials Studio) was used to determine χ_{ij} for different components in the system. These values were then converted to DPD repulsion parameters, a_{ij} via the relation $a_{ij} = 3.27\chi_{ij} + 25$. A single disaccharide moiety was used to describe nitrocellulose beads. Whole molecules were used for neutral salicylic acid (neutral), salicylate (charged), propylene glycol and water beads (water). Furthermore, wall beads (wall) were used and their a_{ij} was arbitrarily set to 999 to indicate that no attraction could take place between the wall and any other beads.

S3 SEM images

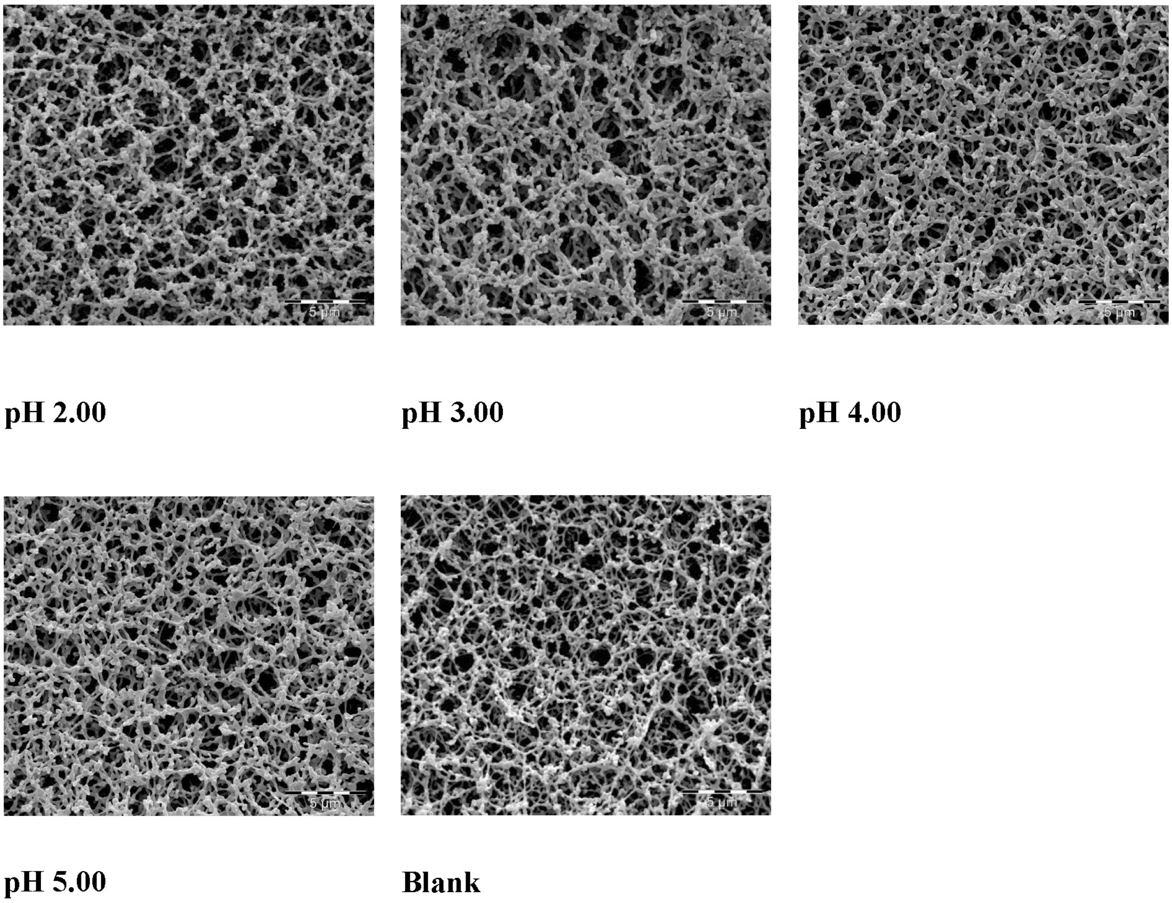

Figure S1. SEM images of nitrocellulose membranes after completion of release study including donor solutions with 1 mg/mL salicylic acid at various pH values and blank-run nitrocellulose membranes. The scale bar in each photograph represents 5 μm.

S4. a_{ij} values of different species at 310 K.

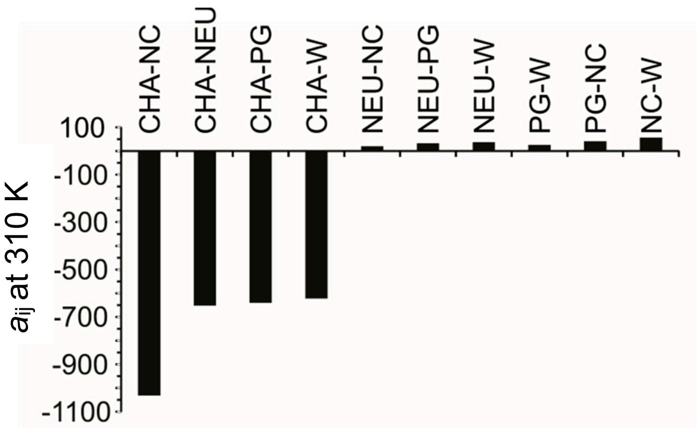

Figure S2. The a_{ij} values calculated for the dissimilar pairs.

S5. χ_{ij} values over the temperature range 273-373 K.

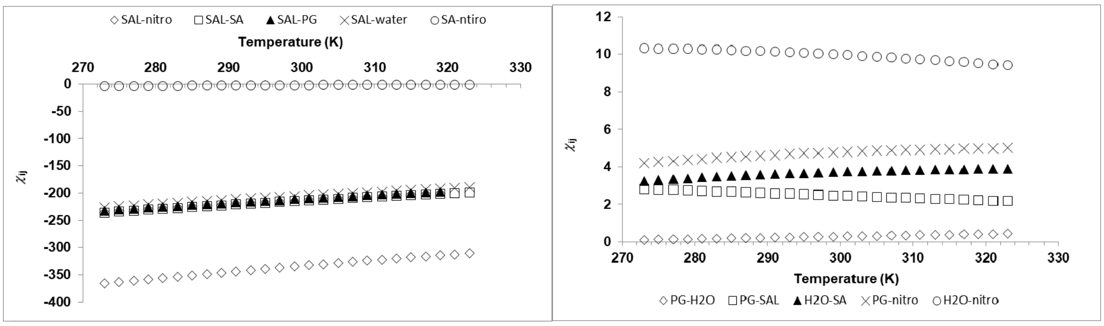

(A) (B)

Figure S3. (**A**) χ_{ij} for miscible pairs at various temperatures. (**B**) χ_{ij} for immiscible pairs at various temperatures. H2O refers to water.

S6. Cohesive energy density (CED)

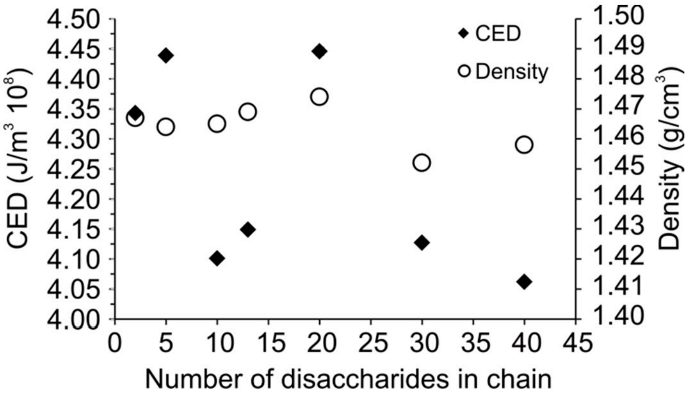

Figure S4. The CED and density values that were obtained for the various chain lengths of nitrocellulose oligomers. The scatter of values is smaller than would be indicated by the depiction if the magnitudes of the axes are inspected.

S7 DPD trajectories of additional models not shown in the main text

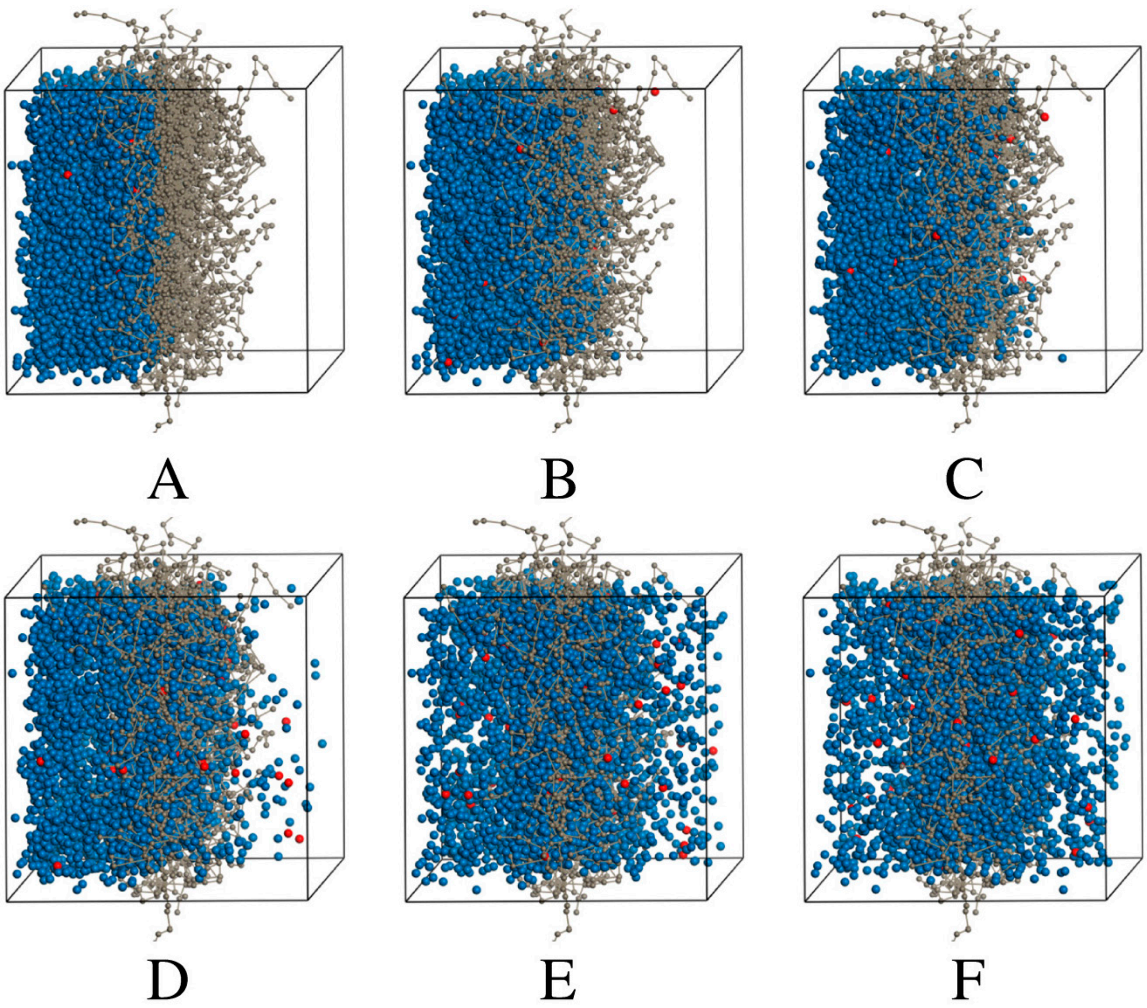

Figure S5. A simulation box of 1:99 (charged:neutral). Nitrocellulose beads (grey), neutral salicylic acid beads (blue) charged salicylate beads (red). Only nitrocellulose, neutral and charged beads are shown at different times as multiples τ of during the trajectory simulation; **A** 1 τ, **B** 12,500 τ, **C** 25,000 τ, **D** 125,000 τ, **E** 500,000 τ and **F** 1,000,000 τ.

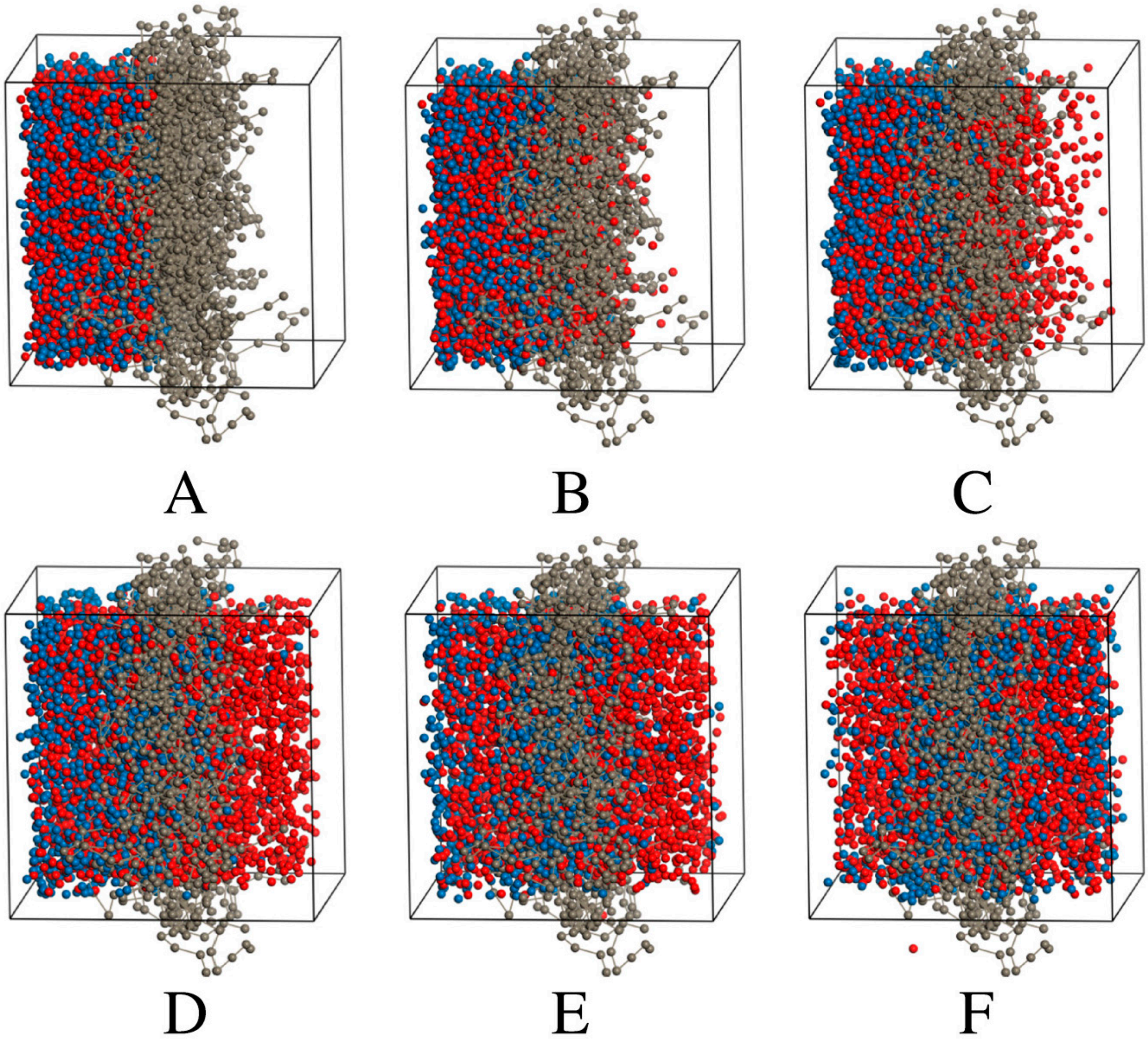

Figure S6. A simulation box (1:1 charged:neutral) with nitrocellulose beads (grey), neutral beads (blue) and charged salicylate beads (red) shown at different times in multiples of τ of during the trajectory simulation; **A** 1 τ, **B** 7,500 τ, **C** 25,000 τ, **D** 125,000 τ, **E** 250,000 τ, **F** 1,000,000 τ.

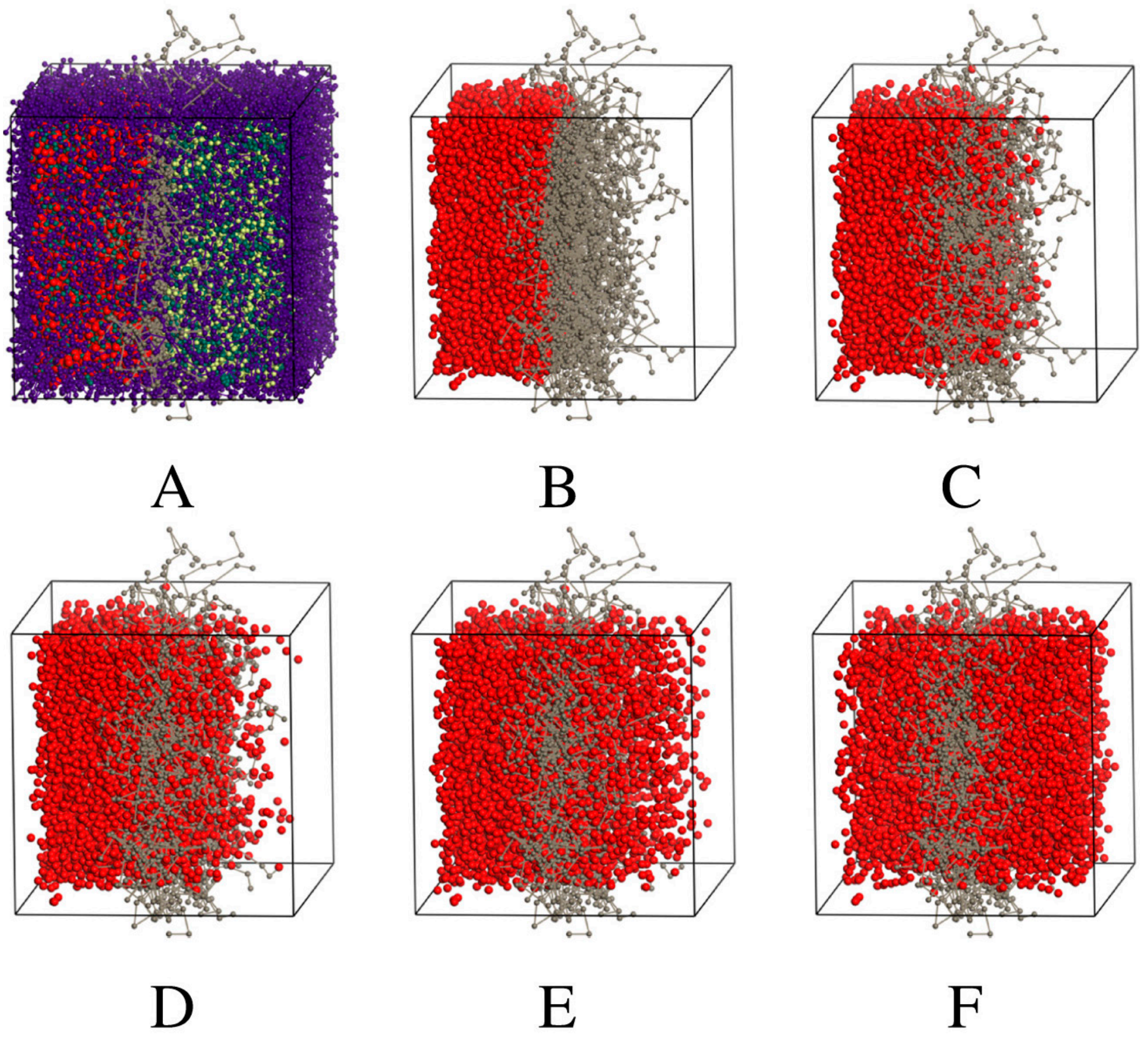

Figure S7. A, the simulation box of 100% charged beads with nitrocellulose beads (grey), wall beads (purple), propylene glycol beads (yellow) and water beads (green) Only charged salicylate and nitrocellulose beads are shown at different times as multiples τ of during the trajectory simulation; B 1τ, C 5,000τ, D 12,500τ, E 25,000τ and F 1,000,000τ.

S8 Propylene glycol concentration profile

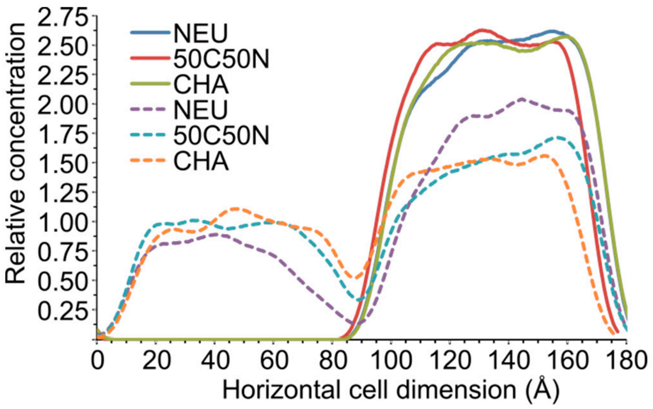

Figure S8. The concentration of propylene glycol as snapshots taken at τ=1 (solid lines) and τ=1,000,000 (dashed lines). C is the abbreviation for charged and N for neutral. The 1:1 system is denoted by 50C50N.

References

Please note that the references are also provided in the main text. The numbering follows the main text.

15. López-Chávez, E.M., Martiínez-Magadán, J.M., Oviedo-Roa, R., Guzmán, J., Ramírez-Saldago, J., Marín-Cruz, J. Molecular modeling of ion-conductivity in chitosan membranes. Polymer, 2005, 46, 7519-7527.
19. Yamane, C., Miyamoto, H., Hayakawa, D., Ueda, K. Folded-chain structure of cellulose II suggested by molecular dynamics simulation. *Carbohydr. Res.* **2013**, *379*, 30-37.
20. Eichhorn, S.J., Young, R.J., Davies, G.R. Modeling crystal and molecular deformation in regenerated cellulose fibers. *Biomacromolecules* **2005**, *6*, 507-513.
21. Ma, X., Zhu, W., Xiao, J., Xia, H. Molecular dynamics study of the structure and performance of simple and double bases propellants. *J. Hazard. Mater.* **2008**, *156*, 201-207.
39. Hoogerbrugge, P.J., Koelman, J.V.M.A. Simulating microscopic hydrodynamic phenomena with dissipative particle dynamics. Europhys. Lett. 1992, 19, 155-160.
40 Koelman, J.V.M.A., Hoogerbrugge, P.J. Dynamic simulations of hard-sphere suspensions under steady shear. Europhys. Lett. 1993, 21, 363-368.
58. Sun, H. COMPASS: An ab initio force-field optimized for condensed-phase applications – overview with details on alkane and benzene compounds. J. Phys. Chem. B 1998, 102, 7338-7364.
67. Fan, C.F., Hsu, S.L. Application of the molecular simulation technique to generate structure of an aromatic polysulfone system, Macromolecules. 1991, 24, 6244-6249.
69. Flory, F.H. Principles of polymer chemistry, Cornell University Press, New York: Ithica, 1953, 672 p.
70. Guo, X.D, Zhang, L.J., Wu, Z.M., Qian, Y. Dissipative particle dynamics studies on microstructure of pH-sensitive micelles for sustained drug delivery, Macromolecules 2010, 43, 7839-7844.
80. Soto-Figueroa, C., Rodríguez-Hidalgo, M., Vincente, L. Mesoscopic simulation of micellar-shuttle pathway of PB-PEO copolymer in a water/[BMIM][PF_6] system. *Chem. Phys. Lett.* **2012**, *531*, 155-159.
84. Srinivas, G., Cheng, X., Smith, J.C. A solvent-free coarse grain model for crystalline and amorphous cellulose fibrils. *J. Chem. Theory Comput.* **2011**, *7(8)*, 2539-2548.
85. Marrink, S.W., Risselada, H.J., Yefimov, S., Tieleman, D.P., de Vries, A.H. MARTINI Force field: coarse grained model for biomolecular simulations. *J. Phys. Chem. B.* **2007**, *111*, 7812-7824.
86. Molinero, V., Goddard, W.A. M3B: a coarse grain force field for molecular simulations of malto-oligosaccharides and their water mixtures. *J. Phys. Chem. B* **2004**, *108* 1414-1427.
87. López, C.A., Rzepiela, A.J., de Vries, A.H., Dijkhuizen, L., Hünenberger, P.H., Marrink, S.J. Martini coarse-grained force field: extension to carbohydrates. *J. Chem. Theory Comput.* **2009**, *5*, 3195-3210.
